# Dopamine Pathway Mediated by DRD5 Facilitates Tumor Growth *via* Enhancing Warburg Effect in Esophageal Cancer

**DOI:** 10.3389/fonc.2021.655861

**Published:** 2021-04-08

**Authors:** Xiaozhe Qian, Donglei Zhang, Ziang Cao, Haitao Ma

**Affiliations:** ^1^ Department of Thoracic Surgery, The First Affiliated Hospital of Soochow University, Suzhou, China; ^2^ Department of Thoracic Surgery, Renji Hospital, School of Medicine, Shanghai Jiaotong University, Shanghai, China

**Keywords:** dopamine pathway, esophageal cancer, Warburg effect, tumor microenvironment, lymphatic metastasis

## Abstract

Esophageal cancer (EC) is among the most malignant cancers globally due to its aggressiveness and poor survival. To set off from the inflammatory tumor immune microenvironment, we analyzed tumor tissues of EC patients with or without lymphatic metastasis to explore the importance of cancer cell derived neurotransmitters. Results have emphasized that the accumulation of dopamine but not other neurotransmitters could be observed in EC tumor tissue of patients, especially those who are bearing lymphatic metastasis. Transcriptional analysis of mentioned tissues was also performed to filter out key enzymes involved in dopamine pathway including tyrosine hydroxylase (TH), DOPA decarboxylase (DCC), monoamine oxidase (MAO), *etc.* Further analysis on tumor tissues of patients indicated that dopamine receptor D5 was aberrantly upregulated and co-located with TH. Both *in vitro* and *in vivo* tests have demonstrated that dopamine could stimulate the proliferation and outgrowth of EC tumor cells *via* the DRD5 mediated pathway. The exploration of mechanism has unveiled that activation of the dopamine pathway significantly enhanced the uptake of glucose and production of lactate of EC tumor cells. It can also facilitate the extracellular acid rate (ECAR), dedicating that DRD5-mediated activated dopamine pathway could effectively form and trigger Warburg effect, which is modulated by the cross-talk of mTOR and AKT pathway. Our results would unveil the relationship between cancer derived neurotransmitters and inflammatory tumor immune microenvironment, thus provide potential therapeutic targets and novel clinical strategy towards metastatic EC.

## Introduction

Esophageal cancer is one of the most fatal diseases and leads to poor prognosis worldwide because of its aggressive clinical course, whose mechanism remains largely unknown (1, 2). According to the “seed and soil” theory, the tumor microenvironment (TME) has been demonstrated to play vital roles in tumor generation (3). Numerous elements will be involved in TME, including tumor associated macrophages (TAMs), cancer associated fibroblasts (CAFs), T cells, and many extracellular matrix (ECM) molecules such as neurotransmitters (4–6). Extensive lines of evidence have pointed out that tumor derived neurotransmitters were vital in tumor growth and metastasis, especially in TME formation and alteration (7, 8). For example, serotonin was reported to promote the proliferation and metabolism of pancreatic ductal adenocarcinoma (PDAC), and acetylcholine (Ach) was also considered to be critical in several kinds of cancers (7, 9). The effects they exerted on tumor or normal cells were totally different from the original ones. While little work has been done to recognize the roles of tumor derived neurotransmitters played in EC inflammatory tumor immune microenvironment, we set off to figure out the mechanism.

Dopamine is well known for its important functions in the nervous system. The key enzyme TH is used to transfer tyrosine to L-dopa, followed by catalysis of DCC to generate dopamine. The degeneration of dopamine required several kinds of enzymes including MAOA, MAOB, and catechol-O-methyltransferase (COMT). The synthesis and degeneration of dopamine are kept balance in normal conditions due to the modulation of expressions of these enzymes.

Here we set off from investigating the tumor derived neurotransmitter levels in tumor tissues of EC. We performed transcriptional analysis on 15 cases of clinical samples including normal tissue, tumor of EC patients without metastasis, and tumor of EC patients with lymphatic metastasis. We have found that TH, the synthetase of dopamine, was overexpressed in tumor tissues, especially those with lymphatic metastasis. Meanwhile, MAOA and MAOB were observed downregulated in these tissues. These discoveries have indicated that the source of dopamine is enriched, while the outlet is abolished. We also found that dopamine was accumulated in the tumor tissue together with one of its receptor DRD5. Both *in vitro* and *in vivo* studies have elucidated that the activation of the dopamine pathway significantly facilitated the proliferation and metastatic ability of EC cells. Moreover, mechanism exploration has demonstrated that this pathway could enhance the Warburg effect for achieving the promotion of cell proliferation and survival. Our study would provide a potential clinical target for EC therapy.

## Materials and Methods

### Clinical Samples

Samples from EC patients mentioned in this study mainly constituted two cohorts for the experimental design. All samples or specimens were from Renji Hospital, Shanghai Jiaotong University School of Medicine. Collections and experimental performance of the process of study were fully approved by the local ethics committee in Renji Hospital. All patients were fully recognized with the consents and were informed.

Cohort I containing 10 EC tissues (5 MT and 5 T) and five adjacent normal tissues (5 N) from patients were used to perform transcriptional analysis (Information in details could be gained in **Supplemental Materials** “**Transcriptional Analysis Information**”).

Cohort II was a group of fresh tissue specimens, consisting of EC primary tumor with or without lymphatic metastasis and adjacent normal tissue. All these patients were well and carefully diagnosed by both clinical surgeons and professional pathologists.

For GSEA, we utilized 4 GB (64 bit) GSEA v4.01 Java Web Start (all platforms). The referred gene set database involved in our study was ftp.broadinstitute.org://pub/gsea/gene_sets/c2.cp.kegg.v7.0.symbols.gmt; permutations number is 1,000; Collapse dataset to gene symbols is “true” and permutation type is “gene_set”.

### Neurotransmitter Concentration Measurement

Dopamine ELISA kit (Cat.: LS-F39204) was purchased from Lifespan Biosciences (Seattle, WA, USA); Serotonin ELISA kit was purchased from Elabscience (Wuhan, China); Choline/Acetylcholine assay kit (ab65345) was purchased from Abcam; Epinephrine ELISA kit (RE59251) and Noradrenalin ELISA kit (RE59261) were purchased from IBL international.

For neurotransmitter concentration measurement, all tissues were washed with the icy PBS for removal of blood and other components. Then they were centrifuged at 13,500 × g for 25 min at 4°C after homogenization. The supernatants of these treated samples were thus carefully removed and transferred for further measurement of neurotransmitters. All measurements were performed following the manufacturer’s instructions.

### Cell Culture and Reagents

Human EC cell lines HEEC, ECA109, EC9706, KYSE30, TE-1, and KYSE140 were preserved at Renji Hospital, Shanghai Jiao Tong University School of Medicine. These cell lines were cultured using DMEM or RPMI1640 medium of 10% fetal bovine serum (FBS). Antibiotic mixture at 1% was used. The culturing of these cell lines was maintained at 37°C with 5% CO_2_. The culturing of cell lines was in biohazard safety equipment and under professional instructions.

### Short-Hairpin and Construction Lentivirus Transduction

In this research, lentivirus carrying short-hairpin RNA targeting TH (5′-GCAGAGGCCATCATGGTAAGA-3′), DRD5 (5′ -GCAGTTCGCTCTATACCAGCA-3′), or Negative control (CON) were all purchased from Genechem Co., Ltd (Shanghai). In overexpression progress, *TH* (NM_199292.3) or DRD5 (NM_000798.5) was performed. The Pglv2 vector was used. Then pPACK package system containing pPACK-GAG, pPAKC-REV, and pPAKC-VSV-G was performed for transfection on 293-T cell lines. Lentivirus carrying specific short-hairpin RNA or genes was then used for cell transfection in the presence of 1× HitranasG transfection reagent (Genechem Co., Ltd). Virally infected cells were thus incubated with 8 μg/ml puromycin (A1113802, Gibco, USA) for selection before the *in vitro* and *in vivo* tests.

### Animal Experiments

In this study, 6–8 week-old male C57BL/6J mice and BALB/C nude mice were all treated with humane care. These mice were fed in accordance with the Guide for the Care and Use of Laboratory Animals prepared by the National Academy of Sciences and published by the National Institutes of Health. All modeling procedures and experimental operations mentioned in this study were approved by the Research Ethics Committee of East China Normal University.

For *in vivo* imaging, 150 mg D-luciferin (Promega) was diluted with 120 μl PBS before intraperitoneal injection of footpad injection in the mouse model. Anesthetization of modeled mice with lymphatic metastasis was achieved by 2.5% vaporized inhaled isoflurane 2 min after injection.

Then mice were placed into *In Vivo* Imaging System (IVIS) Spectrum (Caliper Life Sciences, Waltham, MA). We then detected signals of firefly bioluminescence for tumor metastasis evaluation before auto-quantification.

For subcutaneous xenograft models, KYSE140^CTRL^ and KYSE140^sh^ *^DRD5^* suspended in DMEM at a concentration of 2 × 10^7^ cells/ml were injected; the injecting volume was 200 μl, respectively. Subcutaneous injections of the dual lower limbs of BALB/C nude mice were performed with our prepared cell suspension for implantation of tumor burden. Tumor diameters were monitored with calipers every 4 days until the sacrifice of mice. Tumor volumes were calculated as volume = 0.5 × length × width^2^.

### Western Blotting

For cell lysate preparation, IP-lysate buffer (P0013, Beyotime, China) was used. The inhibition of protein degeneration and dephosphorylation was blocked *via* using protease and phosphatase inhibitor (HY-K0010 and HY-K0023, MCE, China). Centrifugation of lysates for 12 min at 4°C was then performed. Concentrations of contained total proteins in harvested supernatant were then quantified and standardized by BCA Protein Assay Kit (Pierce Biotechnology, USA). Samples at 25 μg/lane were added into the gel of WB and run on 10% SDS-PAGE gel before transferring them to nitrocellulose membranes. For normal proteins, skimmed milk powder (Invitrogen) diluted into TBST (containing 1‰ Tween 20) at a concentration of 5% and for phosphorylated proteins BSA at a concentration of 5% were performed. Primary antibodies were then performed for incubation at 4°C overnight. Horseradish peroxidase (HRP)-conjugated secondary antibody at a dilution ratio of 1:10,000 was performed for probing the proteins on membranes.

Antibodies involved were as follows: HIF-1*α* (D1S7W) Rabbit mAb (#36169), c-Myc (D84C12) Rabbit mAb (#5605), Phospho-Akt (Thr308) (244F9) Rabbit mAb (#4056), Phospho-Akt (Ser473) (587F11) Mouse mAb (#4051), Akt (pan) (11E7) Rabbit mAb (#4685), p70 S6 Kinase (49D7) Rabbit mAb (#2708), Phospho-p70 S6 Kinase (Thr421/Ser424) Antibody (#9204), Phospho-p44/42 MAPK (Erk1/2) (Thr202/Tyr204) (D13.14.4E) Rabbit mAb (HRP Conjugate) (#8544), p44/42 MAPK (Erk1/2) (137F5) Rabbit mAb (#4695), Rb (4H1) Mouse mAb (#9309), Phospho-Rb (Ser807/811) (D20B12) XP^®^ Rabbit mAb (#8516), PRAS40 Antibody (#2610), Phospho-PRAS40 (Thr246) (D4D2)Rabbit mAb (#13175), p-mTOR (#2971), and mTOR (#2983) and were purchased from Cell Signaling Technology (USA).

### Cell Apoptosis Assay

Cell apoptosis was measured by Caspase-3/7 Activity Kit (G7790, Promega): all the cell lines involved in this assay were seeded on 96-well plates at a concentration of 5,000/well. These cells were serum starved for 48 h with or without dopamine treatment before apoptosis examination. The following procedures were strictly performed following the manufacturer’s instructions.

### Immunohistochemistry and Immunofluorescence Staining

Firstly, deparaffinization and rehydration of sections were performed *via* xylene and alcohol treatment. The concentrations of xylene were 100% (20 min)–100% (20 min)–95% (15 min)–85 (15 min)–75% (15 min). Citrate buffer at a temperature of 95°C for 1 min was used for antigen retrieval. After cooling down at room temperature, all tissues on the slides were exposed to 0.3% hydrogen peroxide in methanol to inactivate endogenous peroxidases followed by blockage of BSA at a concentration of 10%. Primary antibody incubation was performed overnight at 4°C (1:200–300).

For IHC-P staining, HRP-conjugated secondary antibody was then used for incubation at room temperature for 2 h. DAB (8059, Cell Signaling Technology, USA) was performed for staining. Counterstaining of hematoxylin for 5 s was then performed.

For IF staining, Triton X-100 (P0096, Beyotime, China) was used on tissue carried slides for 2 min after deparaffinization and rehydration to achieve permeabilization. BSA blocking was then performed.

Primary antibodies were utilized for incubation overnight at 4°C followed by secondary antibody for 2 h at 4°C. The protocols were similar with those in IHC-P staining, from the step of protein marking.

Antibodies involved were as follows:

Anti-Dopamine D1 Receptor antibody (ab81296), Anti-Dopamine D2 Receptor antibody (ab32620), Anti-Dopamine D3 Receptor antibody (ab155098), Anti-Dopamine D4 Receptor antibody (ab135978), Anti-Dopamine D5 Receptor antibody (ab30743), and Anti-Tyrosine Hydroxylase antibody EP1532Y (ab137869) and were purchased from Abcam (USA).

### ECAR Measurement

Diluted tumor cells at 2.5 × 10^4^ per well in a XF-96-well plate to attach overnight before starvation treatment for over 24 h. Unbuffered media were then used for incubation. Sequential administration of the following reagents was performed: 10 mM glucose, 1 mM oligomycin (Sigma-Aldrich), and 80 mM 2-deoxyglucose (2-DG, Sigma-Aldrich, D8375). Seahorse XF96 Flux Analyser (Seahorse Bioscience) was used to measure *in vitro* metabolic alterations. See also our previous study (10, 11).

### Glucose and Lactate Measurement

Tumor cells were planted in six-well pates for measurement after 24 h starvation. Then the supernatants of these cells were collected for further examination. For glucose uptake measurement, glucose assay kit (Sigma-Aldrich) was utilized; for lactate production, Lactate Assay Kit (BioVision) was used. In some experiments, cells were treated with or without Rapamycin (50 nM, Cell Signaling Technology, #9904) before 24 h starvation.

All examinations were performed following the manufacturer’s instructions. See also our previous study (10).

### Quantitative Real-Time PCR

Trizol reagent was used for extraction of total RNA tissues and cells followed by reverse transcription to harvest the cDNA. Real-time PCR on 7500 Real-time PCR system (Applied Biosystems, USA) using the mentioned cDNA was then performed. The primers used in this paper were as follows (**Table 1**).

**Table 1:** Primers of real-time PCR invovled in this study.

Gene	Forward primer (5′ to 3′)	Reverse primer (5′ to 3′)
DDC	TGGGGACCACAACATGCTG	TCAGGGCAGATGAATGCACTG
TH	GGAAGGCCGTGCTAAACCT	GGATTTTGGCTTCAAACGTCTC
DRD5	GGGCAGTTCGCTCTATACCAG	GGTCCAGATGATGAGTAGGGTC
GLUT1	ATTGGCTCCGGTATCGTCAAC	GCTCAGATAGGACATCCAGGGTA
HK2	TGATCGCCTGCTTATTCACGG	AACCGCCTAGAAATCTCCAGA
LDHA	GCTCCCCAGAACAAGATTACAG	TCGCCCTTGAGTTTGTCTTC
PKM	TCGCATGCAGCACCTGATT	CCTCGAATAGCTGCAAGTGGTA
GPI1	CAAGGACCGCTTCAACCACTT	CCAGGATGGGTGTGTTTGACC
PFKL	GGTGCCAAAGTCTTCCTCAT	GATGATGTTGGAGACGCTCA
ALDOA	AACTTTCCTCTGCCTAGCCC	GTACAGGCACAGTCGCAGAG
TPI1	AGCTCATCGGCACTCTGAAC	CCACAGCAATCTTGGGATCT
GAPDH	CTGGGCTACACTGAGCACC	AAGTGGTCGTTGAGGGCAATG
PGK2	AAACTGGATGTTAGAGGGAAGCG	GGCCGACCTAGATGACTCATAAG
PGAM2	AGAAGCACCCCTACTACAACTC	TCTGGGGAACAATCTCCTCGT
ENO1	GCCGTGAACGAGAAGTCCTG	ACGCCTGAAGAGACTCGGT
LDHA	ATGGCAACTCTAAAGGATCAGC	CCAACCCCAACAACTGTAATCT
PDK1	CTGTGATACGGATCAGAAACCG	TCCACCAAACAATAAAGAGTGCT
c-Myc	GTCAAGAGGCGAACACACAAC	TTGGACGGACAGGATGTATGC
HIF-1α	GAACGTCGAAAAGAAAAGTCTCG	CCTTATCAAGATGCGAACTCACA
18S	TGCGAGTACTCAACACCAACA	GCATATCTTCGGCCCACA

### Statistical Analysis

IBM SPSS statistics 19.0 and Graph Pad 7.0 were performed for analysis in this study.

## Results

### Dopamine Is Accumulated at Tumor Tissues of Metastatic EC

**Figure 1:**
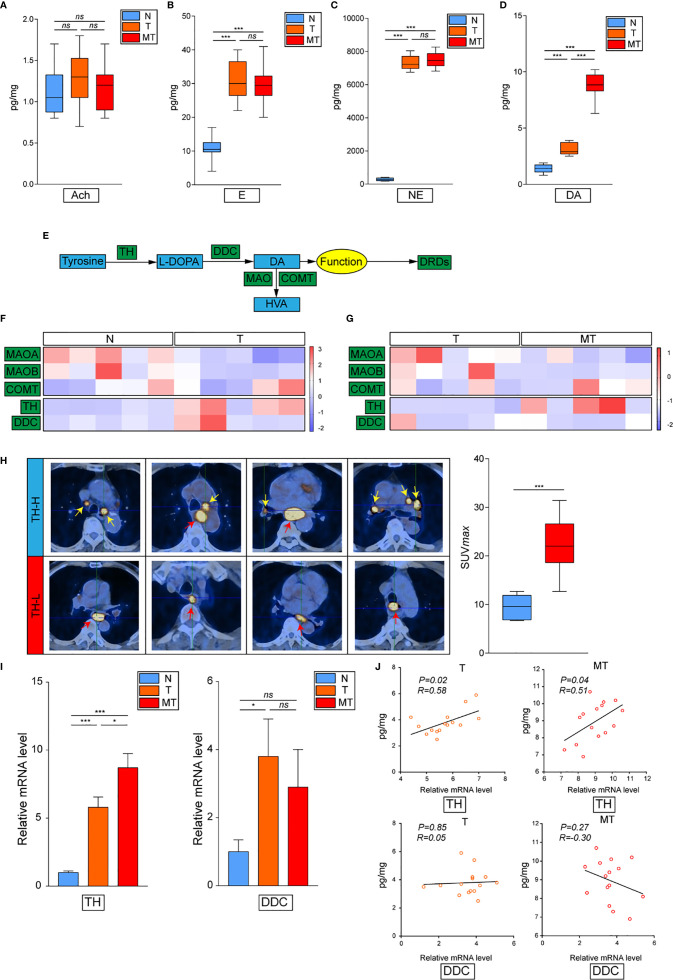
Dopamine is upregulated in EC tissues. **(A–D)** Levels of neurotransmitters including acetylcholine **(A)**, epinephrine **(B)**, norepinephrine **(C)** and dopamine **(D)** were detected by kit in normal esophagus tissue (N), esophageal tumor tissue without lymphatic metastasis (T) or esophageal tumor tissue with lymphatic metastasis (MT) (n = 10 patients per group, three repeats for each sample, mean ± s.e.m.; two-tailed unpaired *t*-test), ***, *P < 0.001, ns. no significant difference*. **(E)** Brief graph displaying the procedure of dopamine metabolism. **(F, G)** Heat map based on RNA-seq showing the relative expressions of key enzymes in dopamine metabolism progress in N *vs* T **(F)** or T *vs* MT **(G)** (n = 5 patients per group). **(H)** PET**–**CT on patients bearing EC showing the relationship of SUV values and TH expression levels (n = 5 patients per group). ***P < 0.001. Arrows: Primary EC, red; lymphatic metastasis, yellow. **(I)** Expressions of TH and DCC were detected by real-time PCR in N, T or MT respectively (n = 12 patients per group, three repeats for each sample, mean ± s.e.m.; two-tailed unpaired *t*-test). *P < 0.05, ***P < 0.001, ns. no significant difference. **(J)** Shown is the relationship between TH or DCC expressions and dopamine levels in T or MT.

To set off, we first collected 30 cases clinical samples from patients bearing EC: adjacent normal esophagus tissue (N), esophagus tumor tissue without metastasis (T), and esophagus tumor tissue with detected lymphatic metastasis (MT). The results have confirmed previous study that cancer derived neurotransmitters could be detected accumulated in tumor tissues. All of E, NE, and dopamine were upregulated in the tumor compared with the normal tissue, while the concentration of dopamine was significantly higher in MT than that in T, indicating that dopamine might play vital roles in inflammatory tumor immune microenvironment (**Figures 1A–D**). The production and metabolism of dopamine involved several key enzymes including the synthetase TH and DDC and its degrading enzymes MAO and COMT (**Figure 1E**). We next performed transcriptional analysis on 15 case clinical samples (five cases N, five cases T, and five cases MT) to evaluate the expressions of these enzymes in EC. The results have demonstrated that the degrading enzymes of dopamine were significantly downregulated in EC tumor, while TH presented significantly higher expression in tumor tissues (**Figure 1F**). Interestingly, the overexpression of TH was higher in MT than that in T, in accordance with the trend of dopamine (**Figure 1G**). Taken together, these phenomena have indicated that the source of dopamine was increased, while the outlet was limited. We then investigated the expression of TH in PET-CT of patients bearing EC with lymphatic metastasis and found that EC with higher TH expression is more prone to generate metastasis (**Figure 1H**). Further results of real-time PCR on clinical samples also consolidated our hypothesis (**Figure 1I**). We next examined the relationship of TH or DCC expression and dopamine concentration in EC and gained that TH and dopamine concentration displayed significant relationship in both T and MT groups (**Figure 1J**). In conclusion, these data have illustrated that dopamine was upregulated in EC and played important roles in lymphatic metastasis.

### DRD5 Is Overexpressed in EC

**Figure 2:**
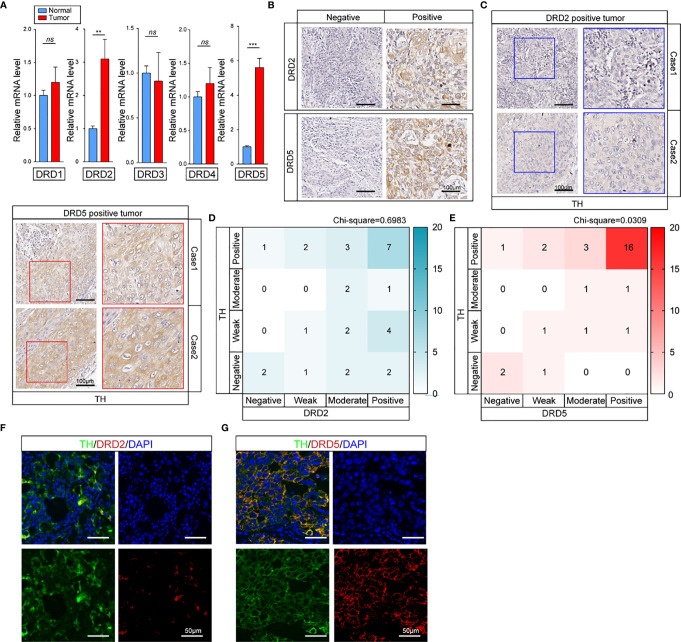
DRD5 is detected to be co-upregulated with TH in EC. **(A)** Expression levels of DRD family (DRD1**–**DRD5) in normal tissues and tumor tissues were examined by real-time PCR (n = 10 patients per group, three repeats per group, mean ± s.e.m.; two-tailed unpaired *t*-test). **P < 0.01, ***P < 0.001, ns. no significant difference. **(B)** IHC-P staining showing DRD2 and DRD5 expressions in EC tumor tissues (n = 30 patients per group, three fields assessed per sample.) Scale bars, 100 μm. **(C)** IHC-P staining of TH on DRD2 or DRD5 positive tumors were performed showing the expression and distribution (n = 30 patients per group, three fields assessed per sample.) Scale bars, 100 μm. **(D, E)** Heat map showing the distribution of TH and DRD2 or DRD5 expressions in EC tissues measured by IHC-P staining (n = 30 patients per group, three fields assessed per sample; independent experiments for each group). **(F, G)** IF staining of TH and DRD2 or DRD5 displaying the co-expression situations IN EC tissues (n = 30 patients per group, three fields assessed per sample.) Scale bars, 100 μm. TH, green; DRD2 or DRD5, red, DAPI, blue.

To further explore how dopamine affects EC progress, we next evaluated the DRD family, which is known to be the most important dopamine receptor. The results elucidated that DRD2 and DRD5 but not other family members, were critical in EC (**Figures 2A, B** and **Supplemental Figure 1A**). Further IHC-P staining has discovered the co-expression of DRD5 but not DRD2 with TH in EC tumor samples (**Figure 2C**). To consolidate, we analyzed the results of IHC-P through scoring and calculated the relationship of DRD2 or DRD5 and TH, which illustrated that DRD5 was co-expressed with TH (**Figures 2D, E**). We also performed IF to observe the colocation of DRD2 or DRD5 and TH; the results further displayed that DRD5 was vital in the dopamine pathway in EC (**Figures 2F, G**). Taken together, these results have shown that DRD5 was aberrantly upregulated in EC tumor cells, which might be responsible for EC growth.

### Dopamine Pathway Promotes Proliferation of Tumor Cells *In Vitro*

**Figure 3:**
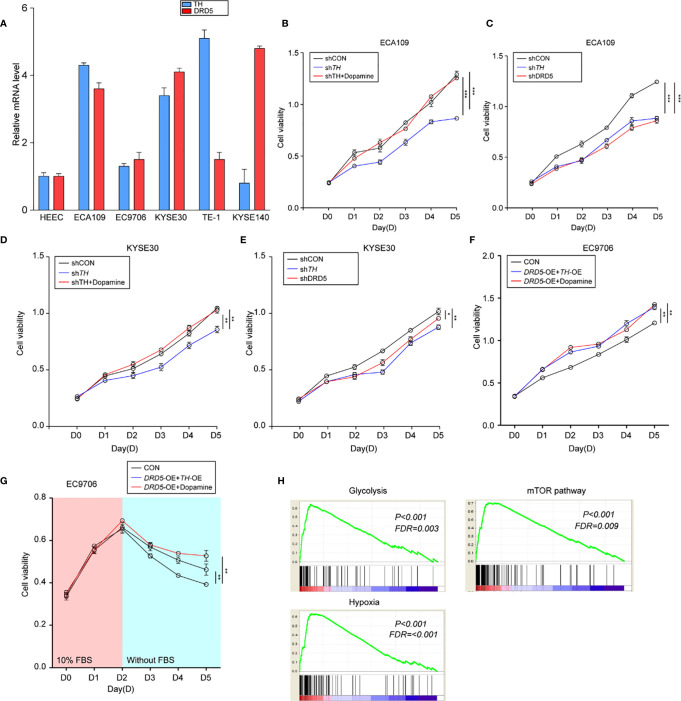
Activation of dopamine pathway promotes tumor cell proliferation and survival of EC. **(A)** Shown is TH and DRD5 expressions in six EC cell lines examined by real-time PCR. **(B–E)** CCK-8 test examined cell viability in ECA109 and KYSE30 cell line. For tests, ECA109 **(B)** and KYSE30 **(D)** cells were performed RNAi on *TH* with or without dopamine administration; or ECA109 **(C)** and KYSE30 **(E)** cells were performed RNAi on both *TH* and *DRD5* for proliferation examination (n = 5 repeats per group, mean ± s.e.m. Repeat measures ANOVA). *P < 0.05; **P < 0.01; ***P < 0.001. **(F)** CCK-8 test examined cell viability in EC9706 cells with dual overexpression of *DRD5* and *TH* or overexpression with dopamine administration (n = 5 repeats per group, mean ± s.e.m. Repeat measures ANOVA). **P < 0.01. **(G)** CCK-8 test examined cell viability in EC9706 cells with dual overexpression of *DRD5* and *TH* or overexpression with dopamine administration in the presence or absence of 10% FBS (n = 5 repeats per group, mean ± s.e.m. Repeat measures ANOVA). **P < 0.01. **(H)** GSEA based on the gene expression profiles of transcriptional analysis. FDR, false discovery rate.

To perform *in vitro* tests, we first evaluated the expression of TH and DRD5 in EC cell lines (**Figure 3A**). It has been shown that the knockdown of TH significantly hampered cell viability through inhibiting the synthesis of dopamine which could be demonstrated by the fact that administration of dopamine could rescue this effect (**Figure 3B** and **Supplemental Figure 1B**). We then proceeded to explore and found that knockdown of either TH or DRD5 would prohibit the proliferation of tumor cells, emphasizing the importance of dopamine pathway in EC (**Figure 3C**). Similar results could also be gained *via* utilizing another cell line KYSE30 (**Figures 3D, E**). We then wondered whether activation of the dopamine pathway through overexpression of key molecules could facilitate proliferation of tumor cells. We next performed dual overexpression of both DRD5 and TH on EC9706 cell line and discovered that this treatment significantly increased cell viability (**Supplemental Figure 1C**). What’s more, the administration of dopamine and overexpression of DRD5 could also lead to the same phenomena (**Figure 3F**). Cell proliferation ability examined by EdU staining has also demonstrated our hypothesis that dopamine facilitated cell viability without inducing significant apoptosis (**Supplemental Figures 1D, E**). The survival ability of tumor cells affected by the dopamine pathway was also taken into consideration. The dual overexpression of which significantly protected the cell from viability decrease in FBS deprivation, demonstrating that this pathway was closely related to survival of tumor cells (**Figure 3G**). We have run GSEA (Gene Set Enrichment Analysis) based on TH expression in our transcriptional analysis and gained that TH was closely related to glycolysis, hypoxia, and the mTOR pathway (**Figure 3H**). To conclude, we have demonstrated that the dopamine pathway positively modulated cell proliferation and growth.

### Dopamine Pathway Facilitates Warburg Effects *via* Mediating Cross-Talk of mTOR and AKT Pathway

**Figure 4:**
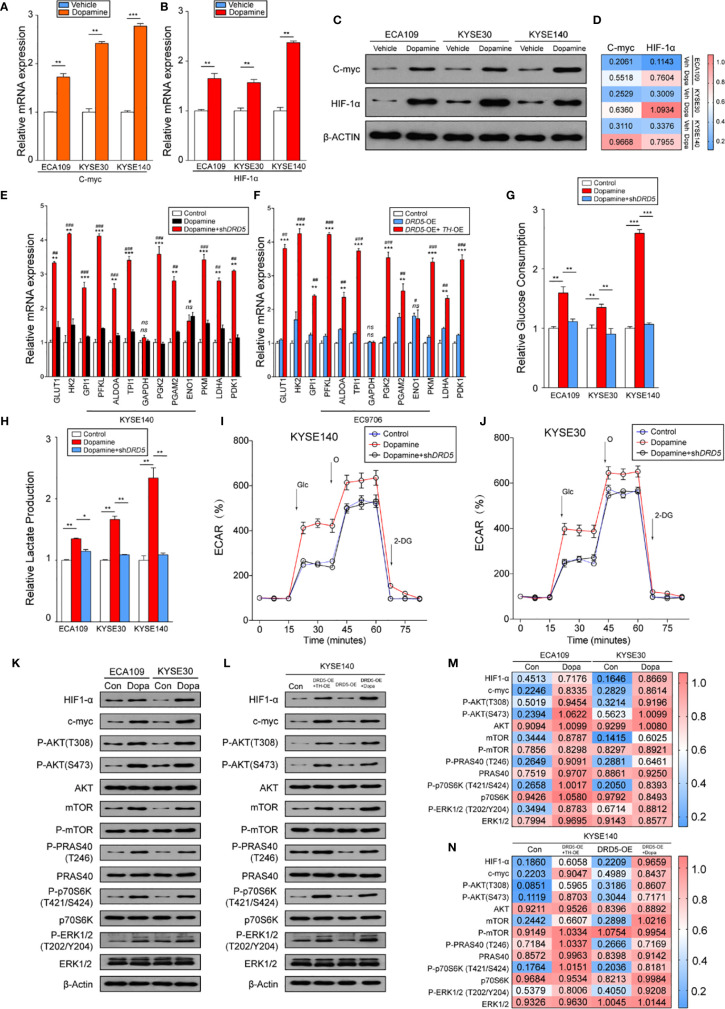
Dopamine promotes Warburg Effect in EC. **(A, B)** Shown is the mRNA level of c-Myc **(A)** and HIF1-*α* **(B)** in EC cell lines after treatment of dopamine. (n = 3 repeats, mean ± s.e.m.; two tailed unpaired t-test). **P < 0.01; ***P < 0.001. **(C, D)** WB assessing c-Myc and HIF1-*α* expression levels in three cell lines after treatment of dopamine. **(E, F)** Relative mRNA levels of glycolytic genes measured in KYSE140 **(E)** or EC9706 **(F)** with or without *DRD5* RNAi in the presence of dopamine stimulation (n = 3 repeats, mean ± s.e.m., two tailed unpaired t-test). #, Control *vs* Dopamine; *, Dopamine *vs* Dopamine + sh*DRD5*; ^*#*^ *P < 0.05*; ^##^ *P < 0.01*; ^###^ *P < 0.001*; *P < 0.05; **P < 0.01; ***P < 0.001; *ns. no significant difference*. **(G, H)** Relative glucose consumption **(G)** or lactate production **(H)** in three EC cell lines with or without *DRD5* RNAi in the presence of dopamine stimulation (n = 3 repeats, mean ± s.e.m., two-tailed unpaired t-test). *P < 0.05; **P < 0.01; ***P < 0.001. **(I, J)** Shown is relative ECARs in KYSE140 **(I)** or KYSE30 **(J)** with or without *DRD5* RNAi in the presence of dopamine stimulation (n = 3 repeats, mean ± s.e.m.). **(K, M)** WB showing the cross-talk of mTOR and AKT pathway mediated by dopamine in two cell lines. **(L, N)** WB displaying cross-talk of mTOR and AKT pathway mediated by dopamine and its key enzymes in KYSE140 cells.

Knowing that the dopamine pathway would promote cell proliferation and survival *via* enhancing glycolysis and hypoxia, we next assessed key gene expressions. Examination of c-myc and HIF-1*α* expression using real-time PCR has displayed that dopamine treatment significantly increased the expression of these two molecules (**Figures 4A, B**). Further consolidation was performed using WB (**Figures 4C, D**). Consistently, the mRNA levels of most metabolic enzymes involved in glycolysis and Warburg effect have shown remarkable elevation after dopamine treatment (**Figures 4E, F**). We also discovered that the activation of dopamine pathway could enhance the relative glucose uptake and promote the extracellular lactate production (**Figures 4G, H**). We have also performed rapamycin, the inhibitor of mTOR pathway, and the results have shown that the administration significantly decreased the glucose consumption and lactate production elevated by dopamine treatment (**Supplemental Figures 1F, G**). To directly assess whether dopamine could stimulate cells to generate the Warburg effect, we examined ECAR of two cell lines *via* utilizing the Seahorse system. The upregulation of ECAR was detected in the treatment of dopamine which was in accordance with our hypothesis. Meanwhile, ECAR would be significantly downregulated once RNAi was performed targeting DRD5, emphasizing the importance of DRD5 in this pathway (**Figures 4I, J**). For mechanism exploration, we have discovered that the dopamine pathway fostered a Warburg effect *via* mediating the cross-talk of AKT and mTOR pathway and thus triggered the ERK–MAPK pathway to activate the expressions of target genes downstream (**Figures 4K–N**). Especially, oncogenic signaling including AKT-p70S6K was also reported in a recent study to take part in Warburg effect.

Collectively, these results have demonstrated that the dopamine pathway could generate a Warburg effect to promote the proliferation and growth in EC.

### Dopamine Pathway Promotes Lymphatic Metastasis and Tumor Cell Outgrowth *In Vivo*

**Figure 5:**
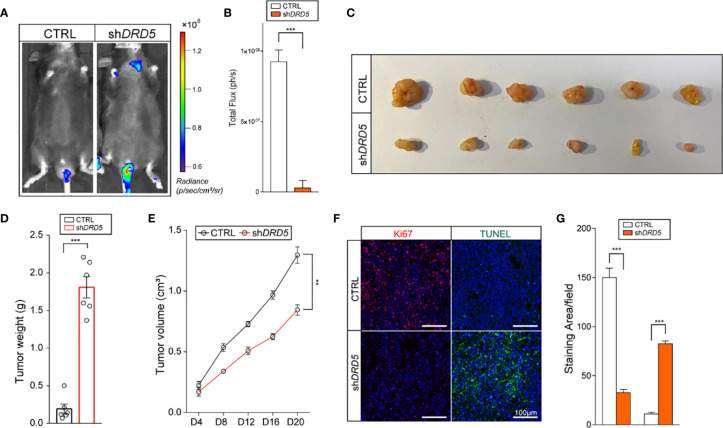
DRD5 mediated dopamine pathway facilitate EC *in vivo.* **(A, B)** Shown is *in vivo* imaging evaluating lymphatic metastasis modeled by footpad injection of EC cells (n = 6 mice per group, mean ± s.e.m.; two-tailed unpaired *t*-test). Scale color bars: 0.60 × 10^8^ **–**1.20 × 10^8^. ***, *P < 0.001*. **(C, D)** Shown is the tumor weights of subcutaneous xenograft models utilizing KYSE140^CTRL^ and KYSE140^sh^ *^DRD5^* (n = 6 mice per group, mean ± s.e.m.; two tailed unpaired *t*-test). ***P < 0.001. **(E)** Tumor growth volumes of subcutaneous xenograft models utilizing KYSE140^CTRL^ and KYSE140^sh^ *^DRD5^* were recorded. (n = 6 mice per group, mean ± s.e.m.; Repeat Measure ANOVA). **P < 0.01. **(F, G)** Representative IF staining of Ki67 and TUNEL performed on subcutaneous xenograft models utilizing KYSE140^CTRL^ and KYSE140^sh^ *^DRD5^* (n = 6 mice per group; three repeats performed per tissue, three fields assessed per sample). Red, Ki67; green dots, TUNEL assay; DAPI, blue. Scale bar: 100μm. ***P < 0.001.

To generate lymphatic metastasis, we performed footpad injection model. In this model, injected tumor cells would generate lymphatic metastasis spontaneously. We have discovered that the knockdown of DRD5 in KYSE140 significantly decreased the metastatic ability to the lymphatic node (**Figures 5A, B**). We have also used the subcutaneous xenograft models. The knockdown of DRD5 significantly attenuated tumor burden of model mice both in tumor weight and tumor growth value (**Figures 5C–E**). Ki67 and TUNEL staining were also performed. In accordance with our formed results, more Ki67 staining could be observed in the control group, while the inference of DRD5 would lead to significant severe apoptosis *in vivo* (**Figures 5F, G**).

In summary, these data illustrated that the knockdown of DRD5 could inhibit the metastasis and growth of tumor cell of EC *in vivo.*

## Discussion

TME consists of different kinds of cells such as aberrantly aggregated immune cells, kinds of chemokines, ECM related molecules, and other secreted proteins. They formed tumor-friendly microenvironments for supporting tumors to grow and metastasize. The key molecules involved in this progress are considered to exert their effects through several functions: 1) directly nourish tumor cells for their proliferation, 2) transfer other native normal cells to special form for tumorigenesis, including forming inflammatory tumor immune microenvironment; 3) protect tumor cells from cell stress such as being attacked by immune cells, low pH microenvironment, *etc.* (12–15). The alteration of TME will also affect the expressions of target genes. In our study, we have demonstrated that TH, acting as the key synthetase of dopamine, was aberrantly overexpressed in EC tumor tissue, causing the accumulation of dopamine. Meanwhile, MAOA and MAOB were detected to be downregulated, meaning that the additional tumor derived dopamine would not be degenerated in time. Following analysis has also confirmed our hypothesis, indicating that the TH overexpression has close relationship with dopamine accumulation. What’s more, we have also noticed DRD5 was also upregulated with TH overexpression and dopamine level elevation. On one hand, enrichment of dopamine could promote growth of DRD5 positive tumor cells, adding survival advantage for tumorigenesis; on the other hand, tumor cells without DRD5 expression would be eliminated. Thus, the dopamine enriched TME provided driving power for tumor cells to survive. Further study has also figured out the co-expression of TH and DRD5 in tumor cells, demonstrating the vital roles of dopamine pathway in EC cells. Results of CCK-8 assay consolidated our hypothesis that the activation of dopamine pathway not only facilitates the proliferation of EC cells, but also prevents tumor cells from apoptosis in FBS removed medium. In proliferation examination, the administration of dopamine could rescue the RNAi of TH, indicating that it was TH synthesized dopamine that played roles in enhancing cell viability of EC. Moreover, dual overexpression of TH and DRD5, but not only one of them, could increase cell viability, demonstrating these two molecules were vital for activating dopamine pathway. *In vivo* tests have fully confirmed our discoveries, both footpad injection model and subcutaneous xenograft models have elucidated the importance of DRD5 in this pathway and might be a potential therapeutic target in dopamine enriched EC.

It has been illustrated that tumor cells prefer to metabolize glucose by aerobic glycolysis rather than through more energetically efficient oxidative phosphorylation, even if the oxygen supply is efficient (16, 17). Moreover, despite the poor efficiency of adenosine triphosphate (ATP) generation, this choice has made it possible for tumor cells in TME to switch glycolytic intermediates into numerous kinds of biosynthetic pathways, which in turn facilitated the biosynthesis of macromolecules required for tumorigenesis or metastasis (18).

Previous studies have revealed that nerve developments have exerted positive effects on cancer (19). Also, cancer cell or TME derived neurotransmitters have been reported to play roles in variant tumors. Both peripheral but not central nervous system derived 5-HT and dopamine could promote outgrowth and metastasis of tumor cells (7, 20, 21). Especially, the promotion effects of 5-HT towards tumor cells were achieved *via* facilitating the Warburg effects (7, 22). In this study, we have discovered the dopamine enrichment at tumor niches in accordance with the expressions of enzymes involved in its metabolism. Further examinations have shown that it was DRD5 but not other DRD family members co-expressed with TH. We thus put forward the hypothesis that autocrine and paracrine of dopamine by tumor cells could stimulate the proliferation through the activation of DRD5.

In mechanism exploration, we have illustrated that the dopamine pathway mediated enhanced cell proliferation, and metastasis would be led by facilitation of the Warburg effect. As aerobic glycolysis is recognized as the most preferred choice for tumor cells rather than oxidative phosphorylation even though abundant oxygen exists, we postulated the dopamine pathway would therefore enhance this progress. Consistently, our results of PET–CT that high expression of TH would lead to high SUV value have also validated this hypothesis. We have also observed that the dopamine pathway activated tumor cells were more able to efficiently uptake glucose and produce lactate, the main production in aerobic glycolysis. Furthermore, we have also detected the activation of AKT-p70S6K pathways and induced expression of HIF-1*α* or c-Myc, the two core molecules against cell stress including hypoxia (23). At last we demonstrated dopamine mediated Warburg effect was based on mediating the cross-talk of mTOR and AKT pathway, which has revealed the importance in this progress (24).

Collectively, both *in vivo* and *in vitro* data have demonstrated that key molecules involved in cancer associated with dopamine pathway might be a promising therapeutic target for patients bearing EC. Targeting DRD5 or TH could achieve better prognosis.

## Data Availability Statement

The datasets presented in this study can be found in online repositories. The names of the repository/repositories and accession number(s) can be found in the article/**Supplementary Material**.

## Ethics Statement

The studies involving human participants were reviewed and approved by No (2017).114. The patients/participants provided their written informed consent to participate in this study. The animal study was reviewed and approved by Research Ethics Committee of East China Normal University.

## Author Contributions

HM was responsible for experiment design. XQ and DZ were responsible for molecular cell and biology experiments. ZC was responsible for animal experiments. HM and XQ were responsible for manuscript writing. All authors contributed to the article and approved the submitted version.

## Funding

This research was supported by the National Key R&D Program of China (2017YFC0114303) and the Natural Science Foundation of Jiangsu Province (BK20191174) hosted by HM.

## Conflict of Interest

The authors declare that the research was conducted in the absence of any commercial or financial relationships that could be construed as a potential conflict of interest.

## Supplementary Material

Supplementary Figure 1 DRD5 meditates proliferation advantage in EC. **(A)** Expressions and protein distributions of DRD family were detected *via* IF staining on EC tissues (three fields assessed per sample). Scale bars, 50 μm. DRD family, green; DAPI, blue. **(B, C)** The inference or overexpression efficiency on EC cell lines was measured by WB. **(D)** Shown is dopamine pathway mediated proliferation activity evaluated by EdU staining (three fields assessed per sample.) Scale bars, 50μm. EdU staining, green; DAPI, blue. **(E)** Shown is the dopamine induced apoptosis in EC cell lines measure by caspase3/7 (n = 5 repeats, mean ± s.e.m., two-tailed unpaired t-test). ns, no significant difference. **(F, G)** Relative glucose consumption **(F)** or lactate production **(G)** in three EC cell lines with or without Rapamycin incubation (50 nmol/L) in the presence of dopamine stimulation (n = 3 repeats, mean ± s.e.m., two-tailed unpaired t-test). *P < 0.05; **P < 0.01.
