# Supplementary figures and images for: Dopamine Pathway Mediated by DRD5 Facilitates Tumor Growth via Enhancing Warburg Effect in Esophageal Cancer
Source: Front Oncol. 2021 Apr 8;11:655861. doi: 10.3389/fonc.2021.655861 (PMC8061419; doi:10.3389/fonc.2021.655861)

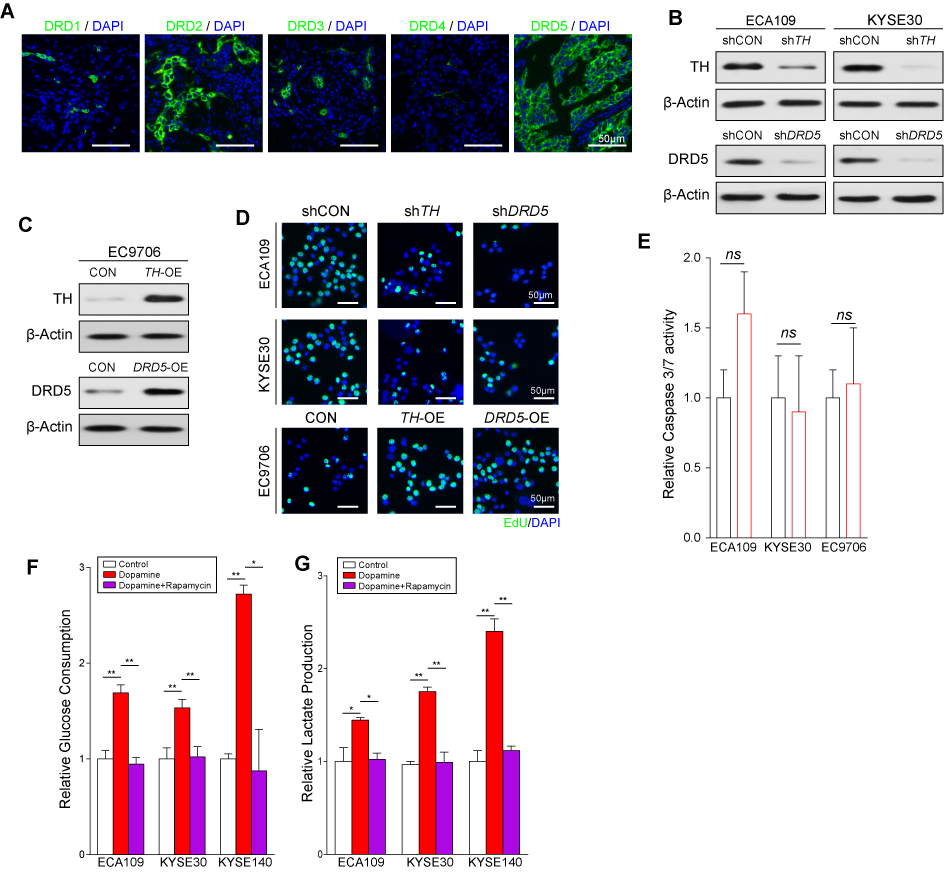

Supplement: Supplementary Figure 1 — DRD5 meditates proliferation advantage in EC. (A) Expressions and protein distributions of DRD family were detected via IF staining on EC tissues (three fields assessed per sample). Scale bars, 50 μm. DRD family, green; DAPI, blue. (B, C) The inference or overexpression efficiency on EC cell lines was measured by WB. (D) Shown is dopamine pathway mediated proliferation activity evaluated by EdU staining (three fields assessed per sample.) Scale bars, 50μm. EdU staining, green; DAPI, blue. (E) Shown is the dopamine induced apoptosis in EC cell lines measure by caspase3/7 (n = 5 repeats, mean ± s.e.m., two-tailed unpaired t-test). ns, no significant difference. (F, G) Relative glucose consumption (F) or lactate production (G) in three EC cell lines with or without Rapamycin incubation (50 nmol/L) in the presence of dopamine stimulation (n = 3 repeats, mean ± s.e.m., two-tailed unpaired t-test). *P < 0.05; **P < 0.01. [file Image_1.tif]
